# Antimicrobial resistance in the Nordics: mapping existing surveillance systems and assessing the impact of COVID-19 using regression models

**DOI:** 10.1186/s13756-025-01552-3

**Published:** 2025-05-28

**Authors:** Tam T. Tran, Adriana Krolicka, Ananda Tiwari, Tarja Pitkänen, Rolf Lood, Ásta Margrét Ásmundsdóttir, Odd-Gunnar Wikmark

**Affiliations:** 1 NORCE Research AS, Nygårdstangen, Bergen, 5838 Norway; 2 Microbiology Unit, Department of Public Health, Finnish Institute for Health and Welfare, Kuopio, 70701 Finland; 3 Department of Food Hygiene and Environmental Health, University of Helsinki, Agnes Sjöbergin Katu 2, Helsinki, 00014 Finland; 4 Department of Clinical Sciences Lund, Division of Infection Medicine, Faculty of Medicine, Lund University, Lund, Sweden; 5 Department of Natural Resource Sciences, University of Akureyri, Akureyri, Iceland; 6 Unit for Environmental Science and Management, Northwest University, Potchefstroom Campus, Private Bag X6001, Potchefstroom, 2520 South Africa

**Keywords:** Antimicrobial use, Antimicrobial resistance, COVID-19, The Nordics

## Abstract

**Background:**

Coronavirus disease 2019 (COVID-19) pandemic constituted the largest global health crisis in recent generations. It may also have disrupted the pattern of antimicrobial use (AMU) and subsequently affected the development of antimicrobial resistance (AMR) – a grave human health concern. This study aimed to give an overview of existing AMR surveillance systems and evaluate the impact of COVID-19 on AMU and AMR in the Nordics using data from these systems.

**Methods:**

Nordic AMU data (2017–2022) were extracted from national annual reports (for both humans and animals) and the European Surveillance System (TESSy) (for humans only). For humans, AMU was expressed in defined daily dose (DDD) per 1000 inhabitants per day; for animals, it was expressed in kilogram (kg). Nordic human AMR data (2017–2022) were extracted from TESSy. Multilevel linear regression and negative binomial regression models were used to fit the TESSy data. Data between 2017 and 2019 were categorised as the pre-COVID-19 time, while data between 2020 and 2022 were the per-COVID-19 time.

**Results:**

Denmark had a remarkably greater AMU in animals (about 10 times greater) than Norway, Sweden, and Finland. Iceland had the highest human AMU, while Sweden had the lowest. Drug categories, countries, and sectors were significant predictors in the model used to fit human AMU. Bacterial species and drug categories were significant predictors in the models used to fit human resistant Gram-negative and Gram-positive bacteria. The COVID-19 time was not a significant predictor in these models. Among the Nordics, Iceland had the lowest number of resistant isolates; however, high human AMU remains a great concern for Iceland.

**Conclusions:**

The study provided insight into current existing AMR surveillance systems in the Nordics. It also showed that the COVID-19 pandemic had very little impact on AMU and AMR in theses countries. This implied that strict regulations on AMU as well as well-coordinated national AMR surveillance systems in the Nordics mitigated the development of AMR crisis also during COVID-19 pandemic. However, the Nordics would still benefit further from a standardized AMR surveillance at regional level, which ultimately facilitate timely information sharing and improve our preparedness for and response to future pandemics and/or large-scale outbreaks.

**Supplementary Information:**

The online version contains supplementary material available at 10.1186/s13756-025-01552-3.

## Background

Coronavirus disease 2019 (COVID-19) was declared a global pandemic by the World Health Organization (WHO) on March 11, 2020 [1]. Since then, COVID-19 has been responsible for an estimated seven million excess deaths up to July 2024 [2]. In addition to these excess deaths, COVID-19 has significant impact on various aspects of everyday lives, including disrupted supply chains, increased food prices and adversely impacted global food security, especially during the early phases [3]. In the Nordics (specifically Norway, Finland, Sweden, Denmark, Iceland and autonomous regions of these countries), the first COVID-19 case was reported in late January 2020 in Lapland Finland, with first confirmed deaths being announced approximately two months later in March simultaneously in Norway and Denmark [4, 6]. Despite the variation in crisis management strategies employed by Nordic countries (i.e. less stringent regulations were implemented in Sweden), the Nordics overall managed the COVID-19 situation relatively well compared to other developed countries, especially in terms of economic repercussions [7].

SARS-CoV-2, the virus responsible for COVID-19, can cause lung complications [8]. Severe cases may present symptoms such as inflammation and fluid buildup, hypoxaemia (below-normal oxygen level in the blood), dyspnoea (shortness of breath). In the early days of the pandemic (before vaccines were publicly available), antibiotics were heavily prescribed to COVID-19 patients to prevent bacterial co-infections [9]. During national lockdowns at the peaks of COVID-19, hospital capacity was often given priority to COVID-19 patients, leading to a decline in hospital admissions for all major non-COVID-19 disease groups [10, 11]. Antibiotics were also used to treat COVID-19 patients with secondary bacterial infections [12]. Therefore, the pattern of antibiotic use and subsequently antimicrobial resistance (AMR) pattern could have changed by the COVID-19 pandemic.

Meanwhile, antimicrobial resistance (AMR) is a multi-faceted global health challenge, also known as a “silent pandemic”. The rate of difficult-to-treat infections caused by resistant pathogens has by far exceeded the rate at which new antimicrobial agents are developed. This is due to the misuse and overuse of antimicrobials, especially those considered drugs of last resort, such as imipenem, meropenem, tigecycline, polymyxin E, daptomycin, vancomycin and linezolid [13, 14]. The death toll associated with AMR was still on the rise, with an estimated 4.95 million deaths in 2019 [15, 16].

To understand the impact of COVID-19 on antimicrobial use (AMU) and AMR in the Nordics, this study will provide an overview of current existing AMR surveillance systems, collect data before and after COVID-19, and apply regression models to these data.

### Nordic countries established national AMR surveillance systems for AMR management

All Nordic countries currently have nationwide AMR surveillance systems in place [17, 20]. They also participate in the European Antimicrobial Resistance Surveillance Network (EARS-Net), which routinely collects data from clinical microbiology/diagnostic/reference laboratories (Table S1). Åland is part of the Finish surveillance system, while the Faroe Islands and Greenland are part of the Danish surveillance system.

Denmark established a surveillance system, named the Danish Integrated Antimicrobial Resistance Monitoring and Research Programme (DANMAP), which was put in place since 1995 by the Danish Ministry of Food, Agriculture and Fisheries and the Danish Ministry of Health [18]. Annual reports cover three categories of bacteria: human and animal pathogens, zoonotic bacteria, and indicator bacteria. The Faroe Islands were included in the DANMAP 2021 report which described the consumption of antimicrobials between 2016 and 2021. Greenland was included in the DANMAP 2022 report which had information on the incidence of multidrug-resistant bacteria between 2000–2022 and the consumption of antimicrobial agents between 2013–2022.

In Finland, the Finnish Study Group for Antimicrobial Resistance (FiRe), a clinical microbiology laboratory network, was established in 1992 and the collection of data started in 1997- the oldest system compared to others in the Nordic area. The legal framework (Communicable diseases act 21.12.2016/1227 and Communicable diseases decree 9.3.2017/146) for AMR surveillance came into operation in 2017. Annual reports of human AMR (Finres-reports), antimicrobial use and AMR in veterinary (FINRES-VET reports) are reported separately [19]. The human sector reporting is in Finnish and veterinary in English.

In Norway, the Norwegian Surveillance System for Antimicrobial Drug Resistance (NORM/NORM-VET) was established in 2000 as a national health registry [17]. In 2003, the registry was regulated according to the Health Registries and Processing of Health Information Act of 18 May 2001. Annual reports provide information on the occurrence of AMR in both human pathogens (NORM), and feed, food and animals (NORM-VET).

Sweden first established a Swedish Veterinary Antimicrobial Resistance Monitoring system back in 2000 [20]. About ten years later, a fully integrated report, a joint production in Human Medicine (Swedres) and Swedish Veterinary Antibiotic Resistance Monitoring (Svarm), started being published annually since 2012.

Iceland also established its nationwide AMR surveillance system and released its first report in 2012 [21]. From 2012 to 2019, the reports were fully in Icelandic; however, there was a supplementary summary in English in the reports since 2020.

### Nordic countries are also participating in the European Surveillance System (TESSy)

The European Centre for Disease Prevention and Control (ECDC), established in 2005 in Stockholm, Sweden, is a public health agency of the European Union (EU) with a vision to improve lives in Europe and globally apply scientific excellence [22]. The ECDC provides TESSy – a platform for EU and European Economic Area (EEA) countries to submit, store, validate and share health-related data. Citizens can access TESSy data through an interactive tool—the Surveillance Atlas of Infectious Disease (Atlas) [23].

The European Surveillance of Antimicrobial Consumption Network (ESAC-Net) was established to collect and analyze data on antimicrobial consumption in humans from EU and EEA countries, both in the community and in the hospital sectors [24]. In July 2011, ESAC-Net was transferred and coordinated by the ECDC. Nordic countries are also part of this network and are coordinated by the following national institutes/organizations: Statens Serum Institut (Denmark), Norwegian Institute of Public Health (Norway), Public Health Agency of Sweden (Sweden), Centre of Health Security and Communicable Disease Control (Iceland), and Finnish Institute for Health and Welfare (Finland).

The European Antimicrobial Resistance Surveillance Network (EARS-Net) was established to collect comparable, representative and accurate AMR data across countries in Europe as well as to provide timely AMR information and intervention [25]. EARS-Net data, which are managed and coordinated by the ECDC, are publicly accessible through the ECDC Surveillance Atlas of Infectious Diseases [23]. Only data from invasive isolates (blood and cerebrospinal fluid) are included in EARS-Net. The national organizations in the Nordics participating in EARS-Net are Statens Serum Institut/Danish Study Group for Antimicrobial Resistance Surveillance (DANRES) (Denmark), University Hospital of North Norway/Norwegian Institute of Public Health/St Olav University Hospital—Trondheim (Norway), Public Health Agency of Sweden (Sweden), National University Hospital of Iceland/Akureyri Hospital/Centre of Health Security and Disease Control (Iceland), and Finnish Institute for Health and Welfare-Department of Health Security/Finnish Hospital Infection Program (SIRO) (Finland).

## Methods

### Data collection

Data on AMU in animals (2017–2022) were extracted from national annual reports. Numerical data from Norway were extracted from a graph using WebPlotDigitizer [26].

Data on AMU in humans (2017–2022) were extracted either from national annual reports or from ESAC-Net.

Data on AMR in humans were obtained from EARS-Net for all five Nordic countries: Denmark, Finland, Iceland, Norway and Sweden. The EARS-Net data contain the following headings: health topic, population, indicator, unit, time, region code, region name, num value. Only data from ‘antimicrobial resistance’ in the health topic for Denmark, Finland, Iceland, Norway and Sweden from 2017 to 2022 were extracted. The data were then parsed into a dataset (used as input data for statistical models) containing following headings: year, country, drug, species, number of resistant isolates, number of total tested isolates.

### Statistical models

Two statistical models used in this study were multiple linear regression and negative binomial regression models. Data used to fit these models were obtained from TESSy because of the consistency and compatibility of the methodology across these Nordic countries.

Data collected in 2017, 2018 and 2019 were considered in pre-COVID-19 time, while data collected in 2020, 2021 and 2022 were considered in per-COVID-19 time.

### Fitting the multilevel linear regression model

A multilevel linear regression model was used to fit AMU data in Nordic countries. The predictors (or independent variables) used in the model included drug categories, countries, sectors and COVID-19 time.

The formula for a multiple linear regression is [27]:

$$y = \beta 0 + \beta 1X1 + \dots + \beta nXn+ \epsilon$$

*y* = the predicted value of the dependent variable

*β*_*0*_ = the y-intercept (value of y when all other parameters are set to 0)

*β*_*1*_*X*_*1*_ = the regression coefficient (*β*_*1*_) of the first independent variable (*X*_*1*_) (or the effect that increasing the value of the independent variable has on the predicted y value)

… = do the same for however many independent variables

*β*_*n*_*X*_*n*_ = the regression coefficient of the last independent variable

*ϵ* = model error (or how much variation there is in our estimate of y)

This model aimed to estimate the relationship between two or more independent variables (drug categories, countries, sectors and COVID-19 time) and one dependent variable (AMU).

### Fitting the negative binomial regression model

A negative binomial regression model was used to fit AMR data in Nordic countries because the count data (number of resistant isolates) were over-dispersed. The data were also divided into two sets: one for Gram-negative bacteria (*Pseudomonas aeruginosa*, *Klebsiella pneumoniae*, *Escherichia coli*, *Acinetobacter* spp.) and one for Gram-positive bacteria *Enterococcus faecalis*, *Enterococcus faecium*, *Staphylococcus aureus*, *Streptococcus pneumoniae*). The predictors used in the model included drug categories, countries, species and COVID-19 time. The number of total tested isolates was used as offset variable in the model.

The formula for negative binomial regression with an offset is [28]:

$$ln\mu = \beta 0 + \beta 1X1 + \dots + \beta nXn + lnt$$

where the predictor variables *X*_*1,*_* X*_*2*_*,…, X*_*n*_ are given, and the population regression coefficients *β*_*0*_*, β*_*1*_*,…, β*_*n*_ are to be estimated; the last term, *lnt* is called the offset.

### Software and packages used for statistical analysis and visualization

All the statistical analyses and visualization were performed with R version 4.3.2 and RStudio 2023.06.2 Build 561.

The packages used for data exploration and cleaning were ‘readr’, ‘dplyr’, ‘tidyr’ [29, 31]. The ‘ggplot2’ and ‘ggh4x’ packages were used to visualize data [32]. The packages used for statistical modeling were ‘rstatix’, ‘performance’ and ‘MASS’ [33–35].

## Results

### AMU in the Nordics

#### AMU in animals

**Fig. 1:**
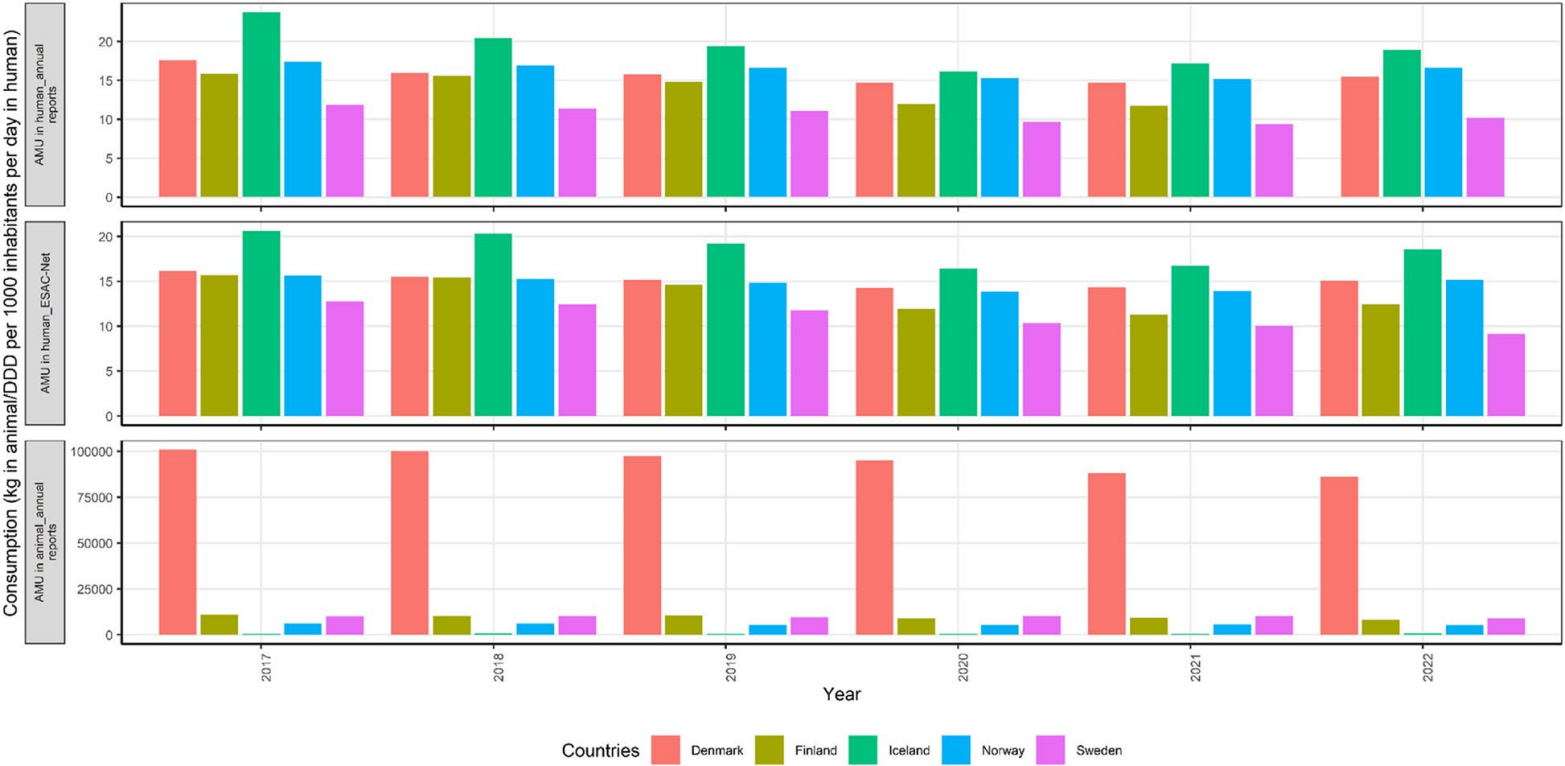
Overall total antimicrobial use (or consumption) in animals (kg) and in humans (Defined Daily Dose (DDD) per 1000 inhabitants per day) for Nordic countries including Denmark, Finland, Iceland, Norway, and Sweden from year 2017 to 2022

**Fig. 2:**
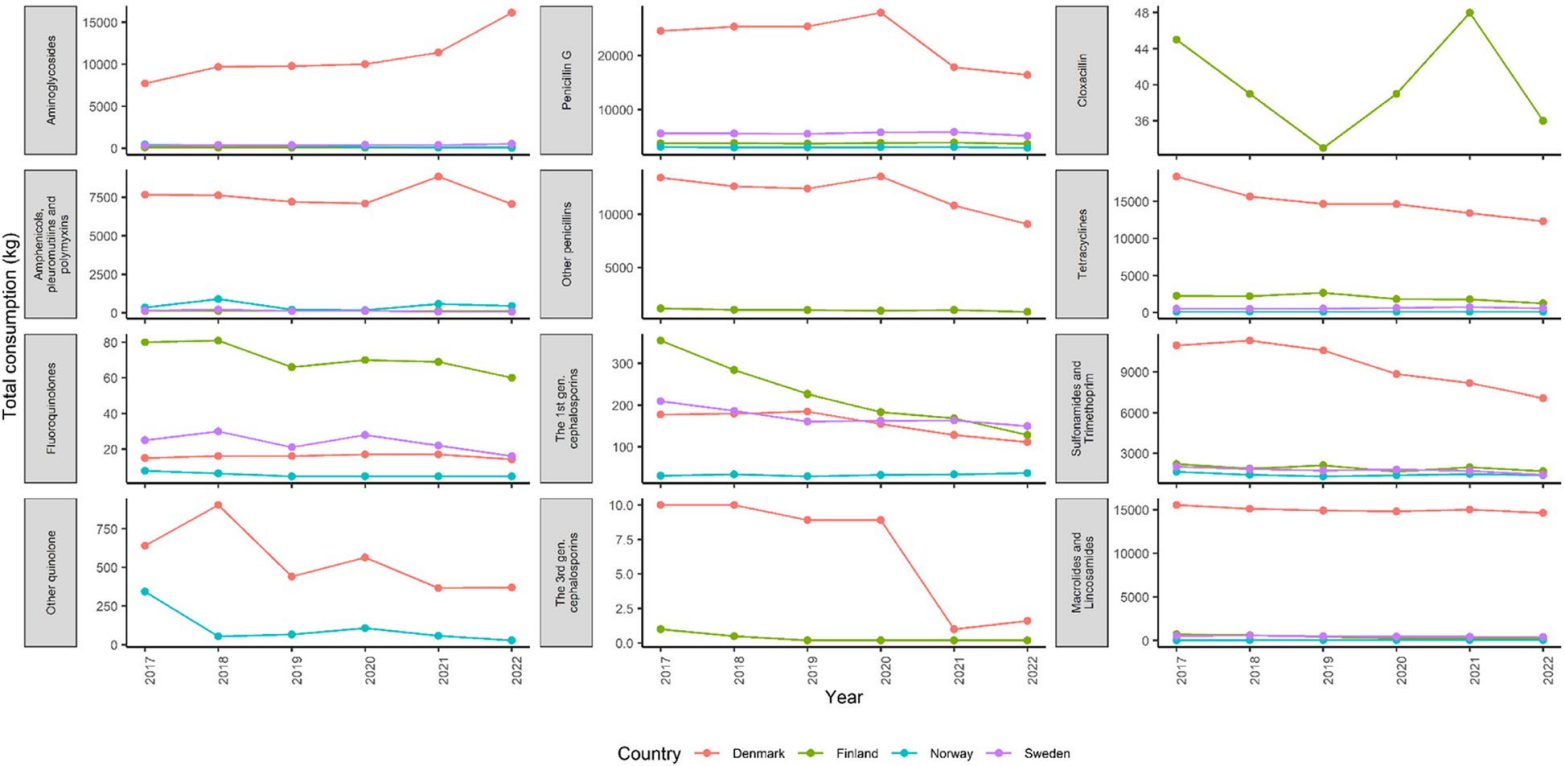
The breakdown of antimicrobial use (or consumption) in animals in Nordic countries including Denmark, Finland, Norway and Sweden from year 2017 to 2022

The data were extracted from national annual reports from all five Nordic countries (Fig. 1). In 2017–2022, Denmark was the country with the most AMU in animals, with AMU approximately ten times greater than that of the other four countries (Iceland, Sweden, Norway and Finland). As a result, Denmark also had the most AMU in most of the drug categories, except for fluoroquinolones and the first generation cephalosporins (Fig. 2). Finland had the highest AMU for fluoroquinolones and the first generation cephalosporins. Only Denmark reported the use of other quinolones, while cloxacillin was used only in Finland. Interestingly, Denmark and Finland were the two countries reporting the use of the third generation cephalosporins which are of important human medicine. However, the use of this drug significantly decreased in Denmark in 2021 and 2022.

#### AMU in humans

Two datasets were collected from two sources for comparison: annual national AMR surveillance reports and ESAC-Net (Fig. 1). Comparing the two datasets of three countries (Denmark, Norway, and Sweden), a negligible difference could be seen, that is, a slightly greater AMU was reported in the reports than in the ESAC-Net database. Iceland consistently had the greatest AMU between 2017 and 2022 while Sweden had the least AMU (data from ESAC-Net).

**Fig. 3:**
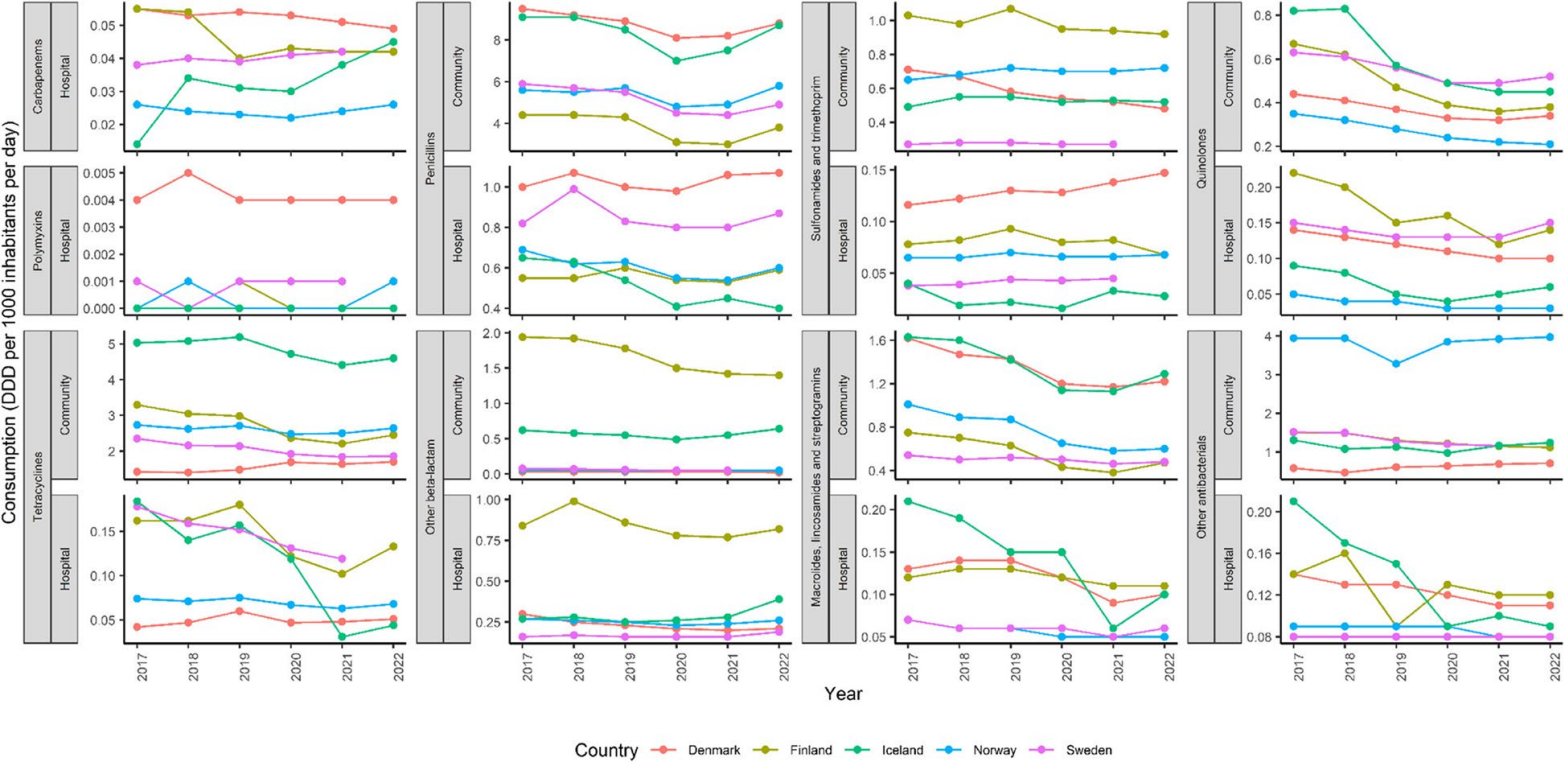
The breakdown of antimicrobial use (or consumption) in humans in Nordic countries including Denmark, Finland, Iceland, Norway and Sweden from year 2017 to 2022. Antimicrobial use is defined as DDD (Defined daily dose) per 1000 inhabitants per day, represented in Y-axis

Carbapenems and polymyxins were allowed to be solely used in the hospital sector (Fig. 3). These drugs are considered last-resort drugs. Denmark had a greater AMU for these two drugs, especially for polymyxins. Denmark also showed outstanding AMU for penicillins and sulfonamides/trimethoprim in the hospital sector. Even though Iceland had the highest total AMU in humans, it only showed substantially higher AMU for tetracyclines in the community sector compared to other Nordic countries. While AMU for carbapenems in hospitals in Iceland showed an upward trend, AMU for tetracyclines, macrolides/lincosamides/streptogramins, and other antibacterial groups in hospitals showed a downward trend. Overall, penicillins and tetracyclines were the two most used drugs in the Nordics (Fig. 3). Cephalosporin and azithromycin in the ESAC-Net database were lumped together with other drugs of the same class, which are cephalosporins and other beta-lactams (J01D) and macrolides, lincosamides and streptogramins (J01 F), respectively. As a result, there was no detailed data on the AMU for these specific drugs; however, the AMU of the drug classes to which they belong ranged from 1.8 to 3 DDD per 1000 inhabitants per day.

According to our multilevel linear regression model of AMU in humans, COVID-19 time did not play a significant role as a predictor of drug consumption (*p*-value > 0.05) (Table 1). However, other predictors, such as drug categories, countries and sectors, were all significant predictors in the model (*p*-values < 0.05). Iceland and Finland had greater mean drug consumption, while Norway and Sweden had less mean drug consumption than Denmark; however, these differences were not statistically significant (*p*-values > 0.05) (Table 2, Table S2). Tetracyclines and penicillins had significantly greater mean drug consumption, while polymyxins had significantly less mean drug consumption than carbapenems (Table 2, Table S3). Interestingly, the hospital sector showed significantly less mean drug consumption than did the community sector (*p*-value < 0.05) (Table 2, Table S3). Pre-COVID-19 time had greater mean drug consumption than per-COVID-19 time, but this difference was not statistically significant (*p*-value > 0.05) (Table 2, Table S3).

**Table 1:** The results of a multilevel linear regression model examining the associations between drug categories, countries, sectors and COVID times on the square root of drug consumption. Analysis of variance (Type II tests)

Response: sqrt(Consumption)				
Variance	Sum Sq	Df	F value	Pr(> F)^**a**^
Drug	66.513	8	75.36	< 2e-16 ***
Countries	1.190	4	2.69	0.03 *
Sectors	60.124	1	544.95	< 2e-16 ***
COVID-19 time	0.299	1	2.71	0.1
Residuals	50.53	458		

^a^Significant codes: 0 ‘***’ 0.001 ‘**’ 0.01 ‘*’ 0.05

**Table 2:** The results of a multilevel linear regression model examining the associations between drug categories, countries, sectors and COVID times on the square root of drug consumption. Coefficients and their *p*-values

Stratification parameters	β	95% CI	*P* value^a^
Intercept	0.91	0.76, 1.06	< 2e-16 ***
Drug categories			
Carbapenems	Referent		
Polymyxins	−0.17	−0.35, −0.002	0.04 *
Tetracyclines	0.4	0.25, 0.55	3.22e-07***
Penicillins	1.08	0.93, 1.23	< 2e-16 ***
Other beta-lactams	−0.01	−0.16, 0.14	0.91
Sulfonamides/trimethoprim	−0.06	−0.21, 0.09	0.44
Macrolides, lincosamides and streptogramins	0.05	−0.1, 0.2	0.54
Quinolones	−0.08	−0.24, 0.07	0.27
Other antibacterials	0.20	0.05, 0.35	0.01 **
Countries			
Denmark	Referent		
Finland	0.06	−0.03, 0.16	0.19
Iceland	0.08	−0.01, 0.18	0.08
Norway	−0.004	−0.09, 0.09	0.93
Sweden	−0.06	−0.15, 0.04	0.25
Sectors			
Community	Referent		
Hospital	−0.76	−0.83, −0.69	< 2e-16 ***
COVID-19 time			
Per-COVID-19 time	Referent		
Pre-COVID-19 time	0.05	−0.01, 0.11	0.1

Residual standard error: 0.3322 on 458 degrees of freedom

Multiple R-squared: 0.7458, Adjusted R-squared: 0.7381

F-statistic: 96 on 14 and 458 DF, *p*-value: < 2.2e-16

^a^Significant codes: 0 ‘***’ 0.001 ‘**’ 0.01 ‘*’ 0.05

### AMR in invasive isolates in the Nordics

#### Gram-negative bacterial resistance

**Fig. 4:**
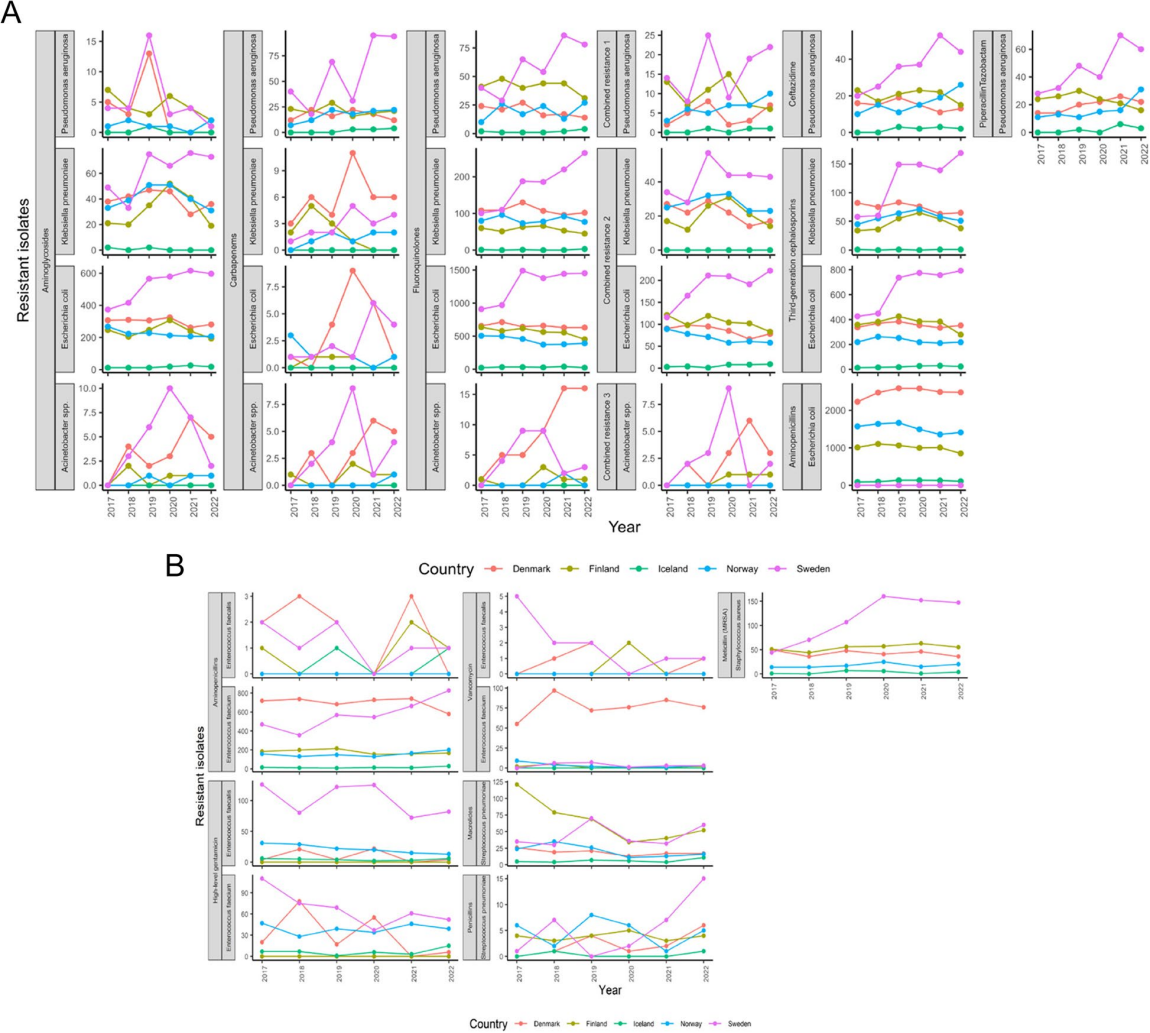
Absolute count data on resistant isolates extracted from the EARS-Net database for Nordic countries including Denmark, Finland, Iceland, Norway and Sweden from year 2017 to 2022. A. Resistant Gram-negative bacteria, B. Resistant Gram-positive bacteria. Combined resistance 1: if isolates that are resistant to at least three of the following drugs: piperacillin-tazobactam, fluoroquinolones, ceftazidime and aminoglycosides. Combined resistance 2: if isolates that are resistant to third-generation cephalosporin, fluoroquinolones and aminoglycosides. Combined resistance 3: if isolates that are resistant to fluoroquinolones, aminoglycosides and carbapenems

Sweden had the greatest number of resistant Gram-negative isolates in most drug categories such as aminoglycosides, carbapenems, fluoroquinolones, ceftazidime, piperacillin-tazobactam and any combined resistance patterns during the 2017–2022 period (Fig. 4A). Denmark had the greatest number of carbapenem-resistant *E. coli* and *K. pneumoniae* as well as aminopenicillin-resistant *E. coli*. Iceland was the Nordic country with the least number of resistant Gram-negative bacteria across all drug categories.

According to our negative binomial model of the number of resistant Gram-negative isolates with offset variable being the total tested isolates, only species and drug categories were significant predictors (*p*-values < 0.05) (Table 3). There were significantly more resistant isolates in Sweden than in Denmark (*p*-values < 0.05) (Table 4, Table S4). There was no significant difference between resistant isolates in the pre-COVID-19 time compared to those in the per-COVID-19 time (*p*-values > 0.05) (Table 4, Table S5). The number of resistant *K. pneumoniae*, *E. coli* or *Acinetobacter* spp. isolates were significantly less than the number of resistant *P. aeruginosa* isolates (*p*-values < 0.05) (Tables 4, Table S5)*.* Isolates resistant to carbapenems or other combined resistance patterns were significantly fewer than those resistant to aminoglycosides (*p*-values < 0.05) (Table 4, Table S5). There were significantly more isolates resistant to fluoroquinolones, third-generation cephalosporins or aminopenicillins than to aminoglycosides (*p*-values < 0.05).

**Table 3:** The results of a negative binomial regression model examining the associations between species, countries and COVID times on the number of resistant Gram-negative isolates with an offset variable being the total tested isolates. Analysis of deviance Table (Type II tests)

Response: NumValue (the number of resistant isolates)			
**Variance**	**LR Chisq**	**Df**	**Pr(> Chisq)**^**a**^
COVID-19 time	0.35	1	0.56
Countries	7.42	4	0.11
Species	22.3	3	5.65e-05 ***
Drug	300.15	9	< 2e-16 ***

^a^Significant codes: 0 ‘***’ 0.001 ‘**’ 0.01 ‘*’ 0.05

**Table 4:** The results of a negative binomial regression model examining the associations between species, countries and COVID times on the number of resistant Gram-negative isolates with an offset variable being the total tested isolates. Resistant Gram-negative isolates

Stratification parameters	β	95% CI	*P* value^a^
Intercept	−2.86	−3.2, −2.52	< 2e-16 ***
Species			
* Pseudomonas aeruginosa*	Referent		
* Klebsiella pneumoniae*	−0.67	−0.97, −0.37	4.56e-08 ***
* Escherichia coli*	−0.49	−0.79, −0.19	4.41e-05 ***
* Acinetobacter* spp.	−0.57	−0.9, −0.24	0.0002 ***
Drug categories			
Aminoglycosides	Referent		
Carbapenems	−1	−1.31, −0.66	< 2e-16 ***
Fluoroquinolones	0.81	0.6, 1.03	5.87e-13 ***
Combined resistance 1	−0.89	−1.34, −0.43	1.73e-05 ***
Combined resistance 2	−0.42	−0.69, −0.15	0.003 *
Combined resistance 3	−0.58	−1.14, −0.03	0.04 *
Ceftazidime	−0.2	−0.63, 0.23	0.29
Third-generation cephalosporins	0.68	0.41, 0.94	1.09e-06 ***
Aminopenicillins	2.4	2.05, 2.76	< 2e-16 ***
Piperacillin-tazobactam	0.04	−0.4, 0.48	0.86
Countries			
Denmark	Referent		
Finland	−0.03	−0.24, 0.18	0.78
Iceland	0.1	−0.16, 0.35	0.45
Norway	0.07	−0.14, 0.29	0.5
Sweden	0.23	0.02, 0.44	0.03*
COVID-19 time			
Per-COVID-19 time	Referent		
Pre-COVID-19 time	−0.04	−0.18, 0.1	0.55

Combined resistance 1: if isolates that are resistant to at least three of the following drugs: piperacillin-tazobactam, fluoroquinolones, ceftazidime and aminoglycosides, Combined resistance 2: if isolates that are resistant to third-generation cephalosporin, fluoroquinolones and aminoglycosides, Combined resistance 3: if isolates that are resistant to fluoroquinolones, aminoglycosides and carbapenems

^a^Significant codes: 0 ‘***’ 0.001 a ‘**’ 0.01 ‘*’ 0.05

#### Gram-positive bacterial resistance

Sweden also had the greatest number of resistant Gram-positive bacteria between 2017 and 2022, especially *E. faecalis* resistant to high-level gentamycin and *S. aureus* resistant to methicillin (Fig. 4B). Denmark had a high number of *E. faecium* resistant to aminopenicillins and *E. faecium* resistant to vancomycin. Similar to AMR in Gram-negative bacteria, Iceland had the least number of resistant Gram-positive bacteria across all drug categories compared to other Nordic countries.

According to our negative binomial model of the number of resistant Gram-positive bacteria with the offset variable being the total tested isolates, species and drug categories were significant predictors in the model (*p*-values < 0.05) (Table 5). Norway and Sweden had significantly fewer resistant isolates than Denmark (Table 6, Table S6). There was no significant difference in the number of resistant isolates in the pre-COVID-19 time compared to those in the per-COVID-19 time (Table 6 Table S7). The number of resistant *E. faecium* isolates was significantly greater, while the number of resistant *S. pneumoniae* was significantly less than the number of resistant *E. faecalis* isolates (Table 6, Table S7). There were significantly fewer isolates resistant to vancomycin, while there were significantly more isolates resistant to high-level gentamicin or macrolide than to aminopenicillins (Table 6, Table S7).

**Table 5:** The results of a negative binomial regression model examining the associations between species, countries and COVID times on the number of resistant Gram-positive isolates with an offset variable being the total tested isolates. Analysis of deviance Table (Type II tests)

Response: NumValue (the number of resistant isolates)			
**Variance**	**LR Chisq**	**Df**	**Pr(> Chisq)**^**a**^
COVID-19 time	0.004	1	0.95
Countries	8.29	4	0.08
Species	209.12	1	< 2e-16 ***
Drug	310.41	3	< 2e-16 ***

^a^Significant codes: 0 ‘***’ 0.001 ‘**’ 0.01 ‘*’ 0.05

**Table 6:** The results of a negative binomial regression model examining the associations between species, countries and COVID times on the number of resistant Gram-positive isolates with an offset variable being the total tested isolates. Resistant Gram-positive isolates

Stratification parameters	β	95% CI	*P* value^a^
Intercept	−3.71	−4.31, −3.1	< 2e-16 ***
Species			
* Enterococcus faecalis*	Referent		
* Enterococcus faecium*	3.25	2.8, 3.7	< 2e-16 ***
* Staphylococcus aureus*	0.05	−0.55, 0.66	0.85
* Streptococcus pneumoniae*	−0.75	−1.39, −0.09	0.006 **
Drug categories			
Aminopenicillins	Referent		
High-level gentamicin	1.1	0.59, 1.62	2.1e-08 ***
Vancomycin	−2.92	−3.36, −2.48	< 2e-16 ***
Macrolides	2.31	1.74, 2.88	< 2e-16 ***
Countries			
Denmark	Referent		
Finland	−0.4	−0.84, 0.03	0.06
Iceland	−0.04	−0.51, 0.43	0.87
Norway	−0.43	−0.85, −0.02	0.03 *
Sweden	−0.43	−0.85, −0.01	0.03 *
COVID-19 time			
Per-COVID-19 time	Referent		
Pre-COVID-19 time	−0.009	−0.27, 0.25	0.95

^a^Significant codes: 0 ‘***’ 0.001 ‘**’ 0.01 ‘*’ 0.05

## Discussion

The One Health continuum recognizes that the environment, animal and human health are interconnected. Therefore, AMU in animals would contribute to the spread of AMR in the environment, which, in turn, poses adverse effects on human health. In general, AMU in animals in the Nordics was still remarkably lower than that in other regions in Europe as well as in the world [36, 37]. Although Denmark had the highest AMU in animals among the Nordics, annual AMU in Denmark was approximately 100,000 kg compared to a few tens of thousands of tons reported in China or Brazil [37]. Denmark is one of Europe’s largest producers of pig meat, which produces close to 33 million pigs annually [38]. Therefore, pig production is the main driver of the consumption of antimicrobials in animals in Denmark (83% of active compounds in 2022). Denmark has also been leading in reducing AMU within that sector. In 2020, the Danish pig sector used 43.3 mg antimicrobials per population correction unit (PCU), in contrast to 68.8 mg per PCU in the Austrian pig sector [38, 39]. A previous study showed that tetracyclines were the most commonly used antimicrobial overall in food-producing animals; however, the AMU intensity per antimicrobial class depended on each country [37]. This finding is in agreement with the Nordic data; that is, tetracyclines were the second most commonly used antimicrobial in Denmark and Finland. Notably, penicillin G was the most commonly used antimicrobial in animals in all Nordic countries. Subclinical concentrations of antimicrobials, especially tetracyclines, have been proven to promote selection and maintenance of multi-drug-resistant bacteria and plasmids [40, 42].

In the Nordics, the two antibiotics most commonly prescribed for human use were penicillins and tetracyclines. This finding was different from what was shown in a previous PRISMA review article, where cephalosporin and azithromycin were the two most commonly prescribed antibiotics [43]. This discrepancy may be because of antibiotic misuse in other countries compared to generally strong regulations on antibiotic use in the Nordics [43]. In our model, human AMU in the Nordics remained nonsignificantly different between pre- and per-COVID-19 time, this may not be the case in other areas of the world. Antibiotic sales were shown to have a positive association with COVID-19 cases at the global scale between 2020 and 2022 [44]. According to the above review study, almost 78% of COVID-19 patients were prescribed antibiotics between 2019 and 2021 [43]. A fourfold increase in the number of antimicrobials used was observed in 2021 compared to that in 2019 [43]. This finding is also in agreement with other studies in which early empiric use of broad-spectrum antibiotics was observed in 79.4% of COVID-19 patients across seven tertiary university hospitals in Croatia, Italy, Serbia and Slovenia and in 71% of hospitalized COVID-19 patients in an adult infectious disease unit in China [45, 46]. However, these studies mainly focused on AMU in hospitalized COVID-19 patients, while the community sector was also included in our study, where a much greater AMU in all drugs where they were allowed was observed compared to that in the hospital sector.

COVID-19 patients may have been misdiagnosed with bacterial pneumonia, such as *S. pneumoniae* or *K. pneumoniae*, due to sharing common symptoms and a lack of effective diagnostic tools [47, 48]. This has led to the misuse of antibiotics for early treatment. The most commonly used anti-pneumococcus agents were β-lactams and macrolides, followed by fluoroquinolone [49]. Penicillins, along with other β-lactams, were also commonly used antibiotics in the Nordics, as mentioned earlier in this study. Denmark had a greater AMU of carbapenems in human medicine, resulting in more carbapenem-resistant *E. coli* and *K. pneumoniae* isolates. Given its relatively high AMU during 2017–2022, Iceland managed to maintain the least number of resistant isolates across all drug categories. Potential factors may include Iceland’s small population size, population density and geographic isolation. Resistant isolates in Iceland were likely underestimated or underreported since the data shown here originated solely from Iceland’s two largest hospitals that were part of the EARS-Net.

Our results here for the Nordics were in line with global data where COVID-19 had less impact on antimicrobial resistance in Gram-positive bacteria [50]. In particular, there was no risk association between COVID-19 and the incidence or proportion of methicillin-resistant *Staphylococcus aureus* or vancomycin-resistant enterococci [50]. There are currently very few studies on the impact of COVID-19 on AMR, especially in Gram-positive bacteria. In those studies, enhanced infection control bundle strategies presumably accounted for a sharp decline in Gram-positive bacterial infections [51, 52]. However, *S. aureus* was found to be among the most common isolates from patients with bacterial coinfections as well as patients with secondary bacterial infections [53].

The Nordic countries have demonstrated a great example of long-term cooperation based on their geographical proximity, similar political ideology, common values and culture of sharing [54]. There are approximately twenty official joint Nordic institutions located in various Nordic countries, and a similar number of unofficial institutions. To name a few, they are the Nordic Innovation Centre (NICe), NordForsk, the Nordic Culture Point, the Nordic Project Fund (NOPEF), the Nordic Centre for Welfare and Social Issues, the Nordic School of Public Health (NHV), the Nordic Cultural Fund, and the Nordic Investment Bank (NIB) [55, 56]. Each Nordic country has its own nationwide surveillance system for AMR. However, there is still a lack of a joint surveillance system for AMR in the Nordics. It has been shown that comparable data obtained from a standardized protocol are essential for tracking the source of outbreaks/pandemics [57, 58]. In this study, the data extracted from the national annual reports of each country were not formatted consistently. Additionally, it was time-consuming and difficult to reformat them, especially for non-English reports. Therefore, we used data from TESSy, specifically ESAC-Net and EARS-Net, to investigate and compare AMU and AMR data in the Nordics. This, however, may also have several limitations such as time delay in data submission, insufficient data curation or missing data due to the large number of participating countries in Europe. Therefore, it may not be tuned for the Nordic context. Our long-term vision is to have a Nordic wastewater-based AMR surveillance network and an integrated digital platform [59]. However, a fundamental Nordic AMR surveillance network that covers AMR data from clinical and/or veterinary settings may need to be established first. This surveillance system can be subsequently extended to include data from environmental surveillance such as wastewater [60]. A joint surveillance system with integrated data across One Health sectors (human health, agriculture and environment) will enhance actionable policy-making decisions [61].

There are a few limitations applying to the scope of this study that are noteworthy. Given the limited data over only six-year span, the outcome in this study should be interpreted with caution. Moreover, selecting the year 2020 to divide data into pre- and per-COVID-19 time may also have some drawbacks. For example, there might have been some delay before the effect happened. Negative binomial regression models only apply on count data, which was the number of resistant isolates in our study. This did not take population size and population density each country into account, and therefore these models may not be the best models to be used for country-wise comparison. However, the model was improved with the offset variable being the total tested isolates. The data from EARS-Net may also introduce some bias into our study as mentioned on the website (i.e. population coverage, sampling and laboratory routines and capacity) [25].

## Conclusion

A detailed overview of all existing AMR surveillance systems in the Nordics was provided in this study. It is currently still lacking consistency in methodology, data collection and formatting among these systems. Our statistical models with current data showed that COVID-19 had very little impact on antimicrobial resistance in the Nordics. This might be a result of strict regulations on antimicrobial use. Nevertheless, Nordics would still benefit further from having a shared Nordic AMR surveillance network.

## Supplementary Information

Supplementary Material 1.

## Data Availability

Data is provided within the manuscript or supplementary information files.
