# Supplementary material for: Antimicrobial resistance in the Nordics: mapping existing surveillance systems and assessing the impact of COVID-19 using regression models
Source: Antimicrob Resist Infect Control. 2025 May 28;14:55. doi: 10.1186/s13756-025-01552-3 (PMC12121021; doi:10.1186/s13756-025-01552-3)
Supplement: Supplementary file 1 — Supplementary Material 1. [file 13756_2025_1552_MOESM1_ESM.docx]

Antimicrobial resistance in the Nordics: mapping existing surveillance systems and assessing the impact of COVID-19 using regression models

Tam T. Tran^1*^, Adriana Krolicka^1^, Ananda Tiwari ^2,3^, Tarja Pitkänen ^2,3^, Rolf Lood^4^, Ásta Margrét Ásmundsdóttir^5^, Odd-Gunnar Wikmark^1,6^

^1^Norwegian Research Centre AS (NORCE), Nygårdstangen, 5838, Bergen, Norway

^2^Finnish Institute for Health and Welfare, Expert Microbiology Unit, Neulaniementie 4, Kuopio FI-70701, Finland

^3^University of Helsinki, Department of Food Hygiene and Environmental Health, Agnes Sjöbergin katu 2, Helsinki FI-00014, Finland

^4^Department of Clinical Sciences Lund, Division of Infection Medicine, Faculty of Medicine, Lund University, Lund, Sweden

^5^Department of Natural Resource Sciences, University of Akureyri, Akureyri, Iceland

^6^Unit for Environmental Science and Management, Northwest University, Potchefstroom Campus, Private Bag X6001, Potchefstroom 2520, South Africa

*Corresponding author: Tam T. Tran, email: tran@norceresearch.no

Table S1: Nationwide AMR surveillance system in the Nordics

| **Country** | **AMR surveillance system in place** | **Established year** | **Voluntary-based** | **Number of participants** | **Part of EARSS/EARS-Net** |
| --- | --- | --- | --- | --- | --- |
| Norway | - Norwegian Surveillance System for Antimicrobial Drug Resistance in human pathogen (NORM) - Norwegian Surveillance System for antimicrobial resistance in animals, food and feed (NORM-VET) | 2000, and regulated in 2003  2000 | Yes     No | All 22 diagnostic + 11 reference laboratories     The Norwegian Veterinary Institute (NVI) and the Norwegian Food Safety Authority (NFSA) | Yes    - |
| Sweden | - Swedish Antimicrobial Resistance Monitoring reports (Swedres-Svarm) - Electronic notification of infectious diseases (Sminet) - Swedish monitoring of antibiotic resistance (Svebar) – a national IT system | 2000  2004  2012 | -  No    Yes | -      22 out of 26 clinical microbiology laboratories (90% population coverage) | -  No    Yes |
| Denmark  The Faroe Islands and Greenland are part of Denmark’s surveillance | - The Danish Integrated Antimicrobial Resistance Monitoring and Research Programme (DANMAP) | 1995 | No | 10 hospital departments of clinical microbiology | Yes |
| Finland  Åland is also part of Finland’s surveillance | - Finnish Study Group for Antimicrobial Resistance (FiRe) - Antimicrobial resistance monitoring reports (Finres) - FINRES-Vet resistance monitoring programme | 1992  1997    2002 | Yes     Yes     Partially | 21 clinical microbiology laboratories and the Bacteriology Unit of the Finnish Institute for Health and Welfare  FiRe group | Yes |
| Iceland | - First report on antimicrobial consumption and antimicrobial resistance in humans and animals | 2012 | - | - | - |

Table S2: Summary of drug consumption data (DDD per 1000 inhabitants per day) collected from ESAC-Net database in the Nordics and categorised by countries from year 2017 to 2022

| Mean and Quartiles | Denmark | Finland | Iceland | Norway | Sweden | All |
| --- | --- | --- | --- | --- | --- | --- |
| Min.  1st Qu.  Median  Mean  3rd Qu.  Max. | 0.004  0.08  0.17  0.95  0.78  9.50 | 0.00  0.12  0.47  0.85  1.08  4.40 | 0.00  0.06  0.39  1.17  0.87  9.1 | 0.00  0.05  0.15  0.92  0.70  5.80 | 0.00  0.06  0.16  0.75  0.80  5.90 | 0.00  0.07  0.24  0.93  0.87  9.50 |

DDD = Defined Daily Doses

Min. = Minimum, Qu. = Quartile, Max. = Maximum

Table S3: The mean drug consumption (DDD per 1000 inhabitants per day) collected from ESAC-Net database in the Nordics by drug categories/sectors/COVID-19 time from year 2017 to 2022

| Drug categories/sectors/COVID-19 time | Mean drug consumption (SD) |
| --- | --- |
| Carbapenems  Polymyxins  Tetracyclines  Penicillins  Other beta-lactams  Sulfonamides and trimethoprim  Macrolides, lincosamides and streptogramins  Quinolones  Other antibacterials | 0.04 (0.01)  0.00 (0.00)  1.45 (1.57)  3.50 (3.16)  0.42 (0.49)  0.35 (0.33)  0.50 (0.50)  0.28 (0.21)  0.87 (1.12) |
| Community  Hospital | 1.90 (2.22)  0.18 (0.24) |
| Per-COVID-19 time  Pre-COVID-19 time | 0.88 (1.61)  0.98 (1.80) |

SD = Standard deviation

Table S4: Summary of the number of resistant Gram-negative isolates (a) and total tested isolates (b) collected from EARS-Net database in the Nordics and categorised by countries from year 2017 to 2022

a.

| Mean and Quartiles | Denmark | Finland | Iceland | Norway | Sweden |
| --- | --- | --- | --- | --- | --- |
| Min.  1st Qu.  Median  Mean  3rd Qu.  Max. | 0.00  5.00  17.50  200.93  93.75  2590.00 | 0.00  2.00  21.00  122.30  64.50  1106.00 | 0.00  0.00  0.00  9.47  3.00  135.00 | 0.00  1.00  18.50  131.90  72.50  1668.00 | 0.00  4.00  33.50  156.40  114.50  1492.00 |

b.

| Mean and Quartiles | Denmark | Finland | Iceland | Norway | Sweden |
| --- | --- | --- | --- | --- | --- |
| Min.  1st Qu.  Median  Mean  3rd Qu.  Max. | 46  431  1142  1993  4883  6017 | 27  381  750  1699  3097  5802 | 0  6.5  25  74.2  192  278 | 23  229  699  1360  3730  4075 | 0  409  851  2627  2161  10633 |

Min. = Minimum, Qu. = Quartile, Max. = Maximum

Table S5: Mean number (and standard deviation) of resistant Gram-negative isolates and total tested isolates collected from EARS-Net database in the Nordics by species/drug categories/COVID-19 time from year 2017 to 2022

| Species/drug categories /COVID-19 time | Resistant Gram-negative isolates | Total tested isolates |
| --- | --- | --- |
| *Pseudomonas aeruginosa*  *Klebsiella pneumoniae*  *Escherichia coli*  *Acinetobacter* spp. | 16.79 (18.22)  40.95 (47.15)  382.58 (551.26)  1.87 (3.07) | 351.41 (220.95)  908.47 (582.20)  4285.37 (2890.47)  50.51 (40.51) |
| Aminoglycosides  Carbapenems  Fluoroquinolones  Combined resistance 1  Combined resistance 2  Combined resistance 3  Ceftazidime  Third-generation cephalosporins  Aminopenicillins  PiperacillinTazobactam | 75.12 (138.22)  7.08 (15.02)  177.26 (322.83)  7.47 (6.47)  56.12 (56.38)  1.10 (2.06)  17.70 (12.63)  193.08 (208.63)  1024.33 (939.18)  21.5 (16.72) | 1489.64 (2383.87)  1495.19 (2384.26)  1513.44 (2394.32)  301.03 (197.70)  2722.50 (2768.34)  49.90 (40.67)  369.80 (230.66)  2755.92 (2804.93)  2525.73 (2189.43)  368.67 (238.87) |
| Per-COVID-19 time  Pre-COVID-19 time | 126.36 (340.50)  122.06 (353.34) | 1676.03 (2590.58)  1425.40 (2092.38) |

Table S6: Summary of the number of resistant Gram-positive isolates (a) and total tested isolates (b) collected from EARS-Net database in the Nordics and categorised by countries from year 2017 to 2022

a.

| Mean and Quartiles | Denmark | Finland | Iceland | Norway | Sweden |
| --- | --- | --- | --- | --- | --- |
| Min.  1st Qu.  Median  Mean  3rd Qu.  Max. | 0.00  2.00  17.00  97.69  53.75  741.00 | 0.00  0.00  2.00  33.93  51.75  214.00 | 0.00  0.00  1.00  3.89  6.00  28.00 | 0.00  1.00  13.50  29.07  28.75  200.00 | 0.00  2.00  35.50  101.40  100.80  828.00 |

b.

| Mean and Quartiles | Denmark | Finland | Iceland | Norway | Sweden |
| --- | --- | --- | --- | --- | --- |
| Min.  1st Qu.  Median  Mean  3rd Qu.  Max. | 0  338  632  693  764  2545 | 0  197  301  534  586  2473 | 13  18.2  30  35.2  35  144 | 64  186  253  464  526  1796 | 0  648  868  1425  1235  7936 |

Min. = Minimum, Qu. = Quartile, Max. = Maximum

Table S7: Mean number (and standard deviation) of resistant Gram-positive isolates and total tested isolates collected from EARS-Net database in the Nordics by species/drug categories/COVID-19 time from year 2017 to 2022

| Species/drug categories /COVID-19 time | Resistant Gram-positive isolates | Total tested isolates |
| --- | --- | --- |
| *Enterococcus faecalis*  *Enterococcus faecium*  *Staphylococcus aureus*  *Streptococcus pneumoniae* | 9.53 (26.13)  122.93 (214.64)  46.23 (43.79)  17.20 (23.45) | 468.87 (435.10)  304.13 (300.28)  2448.50 (2126.59)  452.13 (297.53) |
| Aminopenicillins  High-level gentamicin  Vancomycin  Macrolides  Penicillins  Meticillin (MRSA) | 162.05 (253.73)  27.83 (34.98)  8.82 (23.30)  31.10 (26.62)  3.30 (3.31)  46.23 (43.79) | 454.63 (365.50)  205.02 (326.20)  499.85 (388.60)  455.60 (302.25)  448.67 (297.87)  2448.50 (2126.59) |
| Per-COVID-19 time  Pre-COVID-19 time | 54.36(143.59)  51.87 (127.39) | 663.21 (1187.22)  597.18 (796.82) |
